# Transdermal fluorescence detection of a dual fluorophore system for noninvasive point-of-care gastrointestinal permeability measurement

**DOI:** 10.1364/BOE.10.005103

**Published:** 2019-09-13

**Authors:** Richard B. Dorshow, J. R. Johnson, Martin P. Debreczeny, I. Rochelle Riley, Jeng-Jong Shieh, Thomas E. Rogers, Carla Hall-Moore, Nurmohammad Shaikh, L. Colleen Rouggly-Nickless, Phillip I. Tarr

**Affiliations:** 1 MediBeacon Inc., 1100 Corporate Square Drive, St. Louis, MO 63132, USA; 2 Department of Pediatrics, Washington University School of Medicine, St. Louis, MO 63110, USA

## Abstract

The intestinal mucosal barrier prevents macromolecules and pathogens from entering the circulatory stream. Tight junctions in this barrier are compromised in inflammatory bowel diseases, environmental enteropathy, and enteric dysfunction. Dual sugar absorption tests are a standard method for measuring gastrointestinal integrity, however, these are not clinically amenable. Herein, we report on a dual fluorophore system and fluorescence detection instrumentation for which gastrointestinal permeability is determined in a rat small bowel disease model from the longitudinal measured transdermal fluorescence of each fluorophore. This fluorophore technology enables a specimen-free, noninvasive, point-of-care gastrointestinal permeability measurement which should be translatable to human clinical studies.

## 1. Introduction

Intestinal permeability is increased in multiple intestinal disorders, including Crohn’s Disease, celiac disease, graft vs. host disease, and environmental enteropathy and enteric dysfunction. Permeability is also increased in extra-intestinal disorders such as types I and II diabetes, non-alcoholic fatty liver disease, and juvenile and adult inflammatory arthritidies [1–5]. Interestingly, first degree relatives of people with Crohn’s Disease, who are at increased risk for the development of this disorder, also have increased permeability [6–8].

The prevalence of inflammatory bowel disease (IBD), including Crohn’s disease and ulcerative colitis, is estimated to be 1.2 million people in the US [9], and the total annual economic burden of IBD in the US is estimated to exceed $10 billion [10]. Prevalence of celiac disease is estimated to be 1:70 to 1:300 [11]. The value of earlier detection and anticipatory disease management in such illnesses is substantial [12]. However, current disease monitoring strategies are expensive (colonoscopies), invasive (blood tests), or cumbersome (stool tests).

Intestinal permeability in humans has most commonly been determined using the dual sugar absorption test (DSAT), where patients drink a solution containing a disaccharide, usually lactulose (MW = 342), and a monosaccharide, either mannitol (MW = 182) or rhamnose (MW = 164). These molecules enter the circulation from the gut and are excreted intact into the urine. The urinary ratios of lactulose to mannitol (or lactulose to rhamnose) are used to indicate the degree of intestinal permeability, with increased permeability characterized by an elevated ratio reflecting the heavier disaccharide entering into circulation [13–17]. These DSATs are theoretically sound, but their utility is diminished by the need to collect urine, transport it preserved and frozen to central testing facilities, and the need for sophisticated laboratory instrumentation to measure sugar concentrations for ratio determination [13].

We recently presented data on two fluorescent tracer agents (chosen from our portfolio of renal-excreted fluorophores) of molecular weights similar to the sugars employed in the DSAT [18]. In a gut disease-induced rat model, post oral administration, higher ratios of the larger molecular weight agent to the smaller molecular weight agent were observed for the fluorophores compared to the sugars in the collected urine. These results are graphically shown in Fig. 2 of Reference 18 and also compiled in the supplementary information of that reference.

The two fluorophores also have differing excitation and emission wavelengths. This attribute allows simultaneous transdermal detection of both fluorophores. Hence, a ratio of fluorescent intensity can be constructed in real-time and at the point-of-care. This would permit a specimen-free measurement, overcoming the detrimental attributes of the DSAT.

The report herein describes the transdermal fluorescent detection instrumentation to simultaneously measure each fluorophore, and the companion calibration and analysis. An *in vivo* experiment is performed in a gut disease-induced rat model comparing disease and control results using the fluorescent data. Validation of the transdermal fluorescence measurement results is provided by analysis of the fluorophore content in the collected urine.

## 2. Materials and methods

### 2.1. Fluorophores

**Fig. 1.**
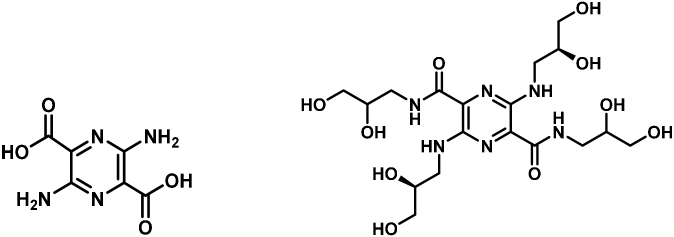
MB-301 (left) and MB-404 (right) structural diagrams.

Motivation for this study is due, in part, to the excretion pathway of the chosen fluorescent tracer agents. These fluorophores are excreted by glomerular filtration, as are the typical sugars employed in the standard DSAT. The two fluorophores in this study, both belonging to the pyrazine class of compounds, were chosen to approximately match the molecular weights of the dual sugar combination lactulose and mannitol. The structures are shown in Fig. 1

.

MB-301 (3,6-diaminopyrazine-2,5-dicarboxylic acid) has a molecular weight of 198, with light absorption and emission maxima at wavelengths 405 nm and 540 nm, respectively [19]. MB-404 (N2,N5-bis(2,3-dihydroxypropyl)-3,6-bis(((S)-2,3-dihydroxypropyl)amino)pyrazine-2,5-dicarboxamide) has a molecular weight of 492, with light absorption and emission maxima at wavelengths 500 nm and 620 nm, respectively. In animal experiments, this compound is excreted almost entirely by the renal system, with negligible protein binding [20]. Further properties of this agent are listed in Reference 20 under the identifier of compound **2h**.

### 2.2. Animals

The Animal Studies Committee of the Washington University School of Medicine approved the animal protocol. Indomethacin-induced small bowel injury in female Sprague-Dawley rats (Charles River, Kingston, NY) was used as the model [21–27]. Indomethacin (Sigma, St. Louis, MO) was suspended in 2% methylcellulose (Sigma, St Louis, MO) in water, at a concentration of 3.75 mg/mL.

### 2.3. Experimental set-up and procedures

On the day prior to the oral tracer gavage, 4 mL/kg of the indomethacin solution vehicle was administered via gavage (approximately 18-20 hours prior to oral tracer gavage) to induce gut lesions in the designated rats. This corresponded to a challenge dose of 15 mg/kg. Vehicle alone (2% methylcellulose) was used for the non-injury control rats, also administered at a volume of 4 mL/kg. Following either of these gavages, a 2.5 × 2.5 cm area on the anterior section of each animal’s back was shaved and treated with depilating cream, necessary for the transdermal experiments described below.

On the following day (day of the oral tracer gavage), animals were anesthetized with 1.5% Isofluorane, and a catheter inserted into their urinary bladder. The fluorophore tracer gavage mixture was prepared consisting of 46 mg/mL MB-404 and 8 mg/mL MB-301, and administered at 2 mL/kg yielding a dose of 92 mg/kg MB-404 and 16 mg/kg MB-301.

**Fig. 2.**
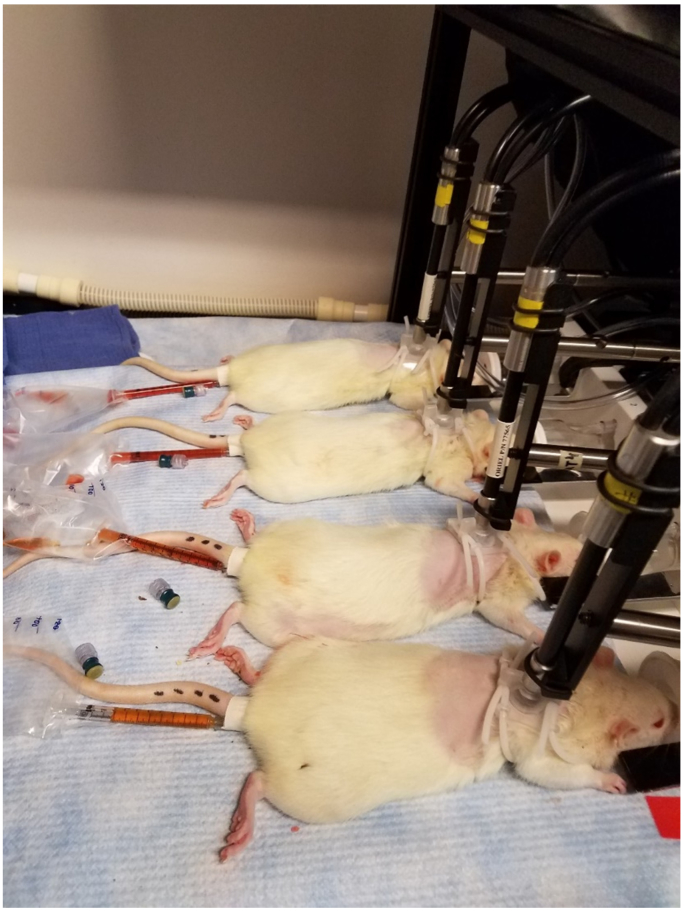
Experimental set-up for *in vivo* transdermal fluorescence acquisition and urine collection.

The anesthetized animals were placed prone (Fig. 2

) and the depilated area was wiped with a ChloraPrep One-Step (CareFusion, 260299) skin prep applicator, followed by wiping with a Cavilon (3M 3344) applicator. A 2.5 × 2.5 cm medical adhesive (3M 1577) was placed over the prepped area. A bifurcated fiber optic bundle for light excitation delivery and fluorescence emission collection, outfitted with a Vascular Access Harness (Instech Labs CIH105), was gently placed against shaved area. A holding arm for the fiber optic bundles (located to the right of each bundle in Fig. 2) was used to support the weight of the cable.

Transdermal fluorescence detection was initiated and baseline fluorescence was acquired for 10 minutes. Animals were then removed from the anesthesia holder for vertical gavage with the fiber optic bundle remaining attached. The animals were placed into the anesthesia manifold following gavage. Animals were gavaged sequentially.

### 2.4. Collection and analysis of fluorophores in urine

Urine was obtained before fluorophore gavage (T_0_), and then at 30, 60, 120, 180, 240, 300, 360, 420, and 480 minutes after fluorophore gavage. Urine volumes were recorded at each time point and pooled together in a tube for the entire period of experiment for each animal in this study. Samples were cooled in an ice bucket before freezing (−80 °C), and remained frozen until analysis.

MB-301 and MB-404 concentrations in urine were determined using a Waters Alliance 2695 HPLC system, or a Waters Acquity H Class UPLC system, equipped with column heater, sample heater/cooler, vacuum degasser, autosampler, binary gradient pump, and fluorescence detector. A Phenomenex Luna C18 (2), 4.6 × 250 mm, 5 µm, 100Å RP-HPLC analytical column (Phenomenex, Cat No. 00G-4252-E0, S/N H15-133556) with a Security Guard Cartridge C18 (4 × 3 mm ID, 5 µm) (Phenomenex, Cat. No. KJ0-4282) were employed for urine sample fluorescence analysis under the HPLC gradient conditions described as follows: Two mobile phases, A: 0.1% TFA/H_2_O and B: 0.1% TFA/ACN were used. The run started with 3 minutes equilibration at 95A/5B, then linear gradient to 60A/40B from 3–10 minutes to separate MB-301 and MB-404. The column was regenerated at 10A/90B from 10.05–12 minutes and at 95A/5B from 12.05–15 minutes.

On the day of analysis, urine samples were thawed to room temperature, and vortexed at high speed for 10 seconds. Ten µL of urine samples were diluted into 990 µL of 1X PBS in amber HPLC vials, mixed thoroughly via vortex mixer, and placed in the autosampler at 5°C. A 480 nm excitation and 600 nm emission wavelength combination was used for MB-404 and MB-301 fluorescence quantitation. Ten µL of sample was injected per run. Two calibration standard sets, one for MB-301 and the other for MB-404, were prepared from dosing solutions. Calibration standards ranged from 9.9 µg/mL to 396.3 µg/mL for MB-301, and 24.6 µg/mL to 985.0 µg/mL for MB-404. MB-301 and MB-404 quality control samples were prepared at 237.8 µg/mL and 591.0 µg/mL respectively. All standards, quality control samples, and study samples were prepared in the same manner, except that standards and quality control samples were diluted 100x in 1% urine in PBS. A linear regression fitting was made with 1/X weighing using the Empower-3 software associated with Waters HPLC and UPLC system.

### 2.5. Transcutaneous fluorescence instrumentation

**Fig. 3.**
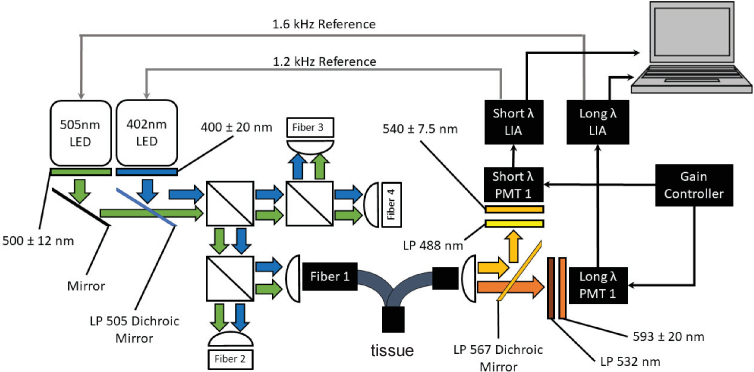
Transdermal fluorescence detection system schematic illustrating the illumination system and one of four identical detection channels. (Filter center wavelengths given with full width at half maximum bandwidth (FWHM).)

A dual-wavelength transdermal fluorescence detection system was constructed to simultaneously monitor MB-301 and MB-404 *in vivo*. The system consisted of four identical detection channels, enabling simultaneous monitoring of up to four animals. Figure 3

illustrates the illumination system, one of the four bifurcated fiber bundles, and one of the four detection channels. Fiber bundles 2–4 and detection channels 2–4 have been omitted for the sake of simplifying the diagram but were identical in their implementation to fiber bundle 1 and detection channel 1. The common end of all four fiber bundles is visible in Fig. 2.

The illumination sources were a 402 and a 505 nm light-emitting diode (LED) (Lightspeed Technologies Inc., Model LEDH-402 and LEDH-505), each packaged in a driver with integrated optics (Lightspeed Technologies Inc., Model HPLS-36AD3500). LED emission was collimated (Lightspeed Technologies Inc., Model OH36-24 × 11) before passing through a 40 nm single bandpass filter centered at 400 nm (Semrock FF01-400/40-25) or a 24 nm single bandpass filter centered at 500 nm (Semrock FF01-500/24-25). LED output was then diverted 90° into a 50:50 beam splitter via a standard mirror (505 nm LED) or a 505 nm long pass dichroic mirror (ThorLabs DMLP505R) (402 nm LED). Resulting LED outputs were split again via two 50:50 beam splitters resulting in four equal dual-wavelength light sources. Each resulting output was focused onto one end of a bifurcated fiber bundle (Oriel 77565), only one of which is depicted in Fig. 3. Fiber bundles consisted of a randomly-mixed, close-packed array of source and detection fibers (individual fiber diameter: 55 µm, 0.56 NA, common bundle diameter: 4.5 mm, total bundle length: 2 m). Light level was controlled by adjusting the amplitude of a 1.2 kHz (402 nm LED) or 1.6 kHz (505 nm LED) square-wave modulated LED driver signal from a Stanford Research SR-830DSP lock in amplifier. Irradiance at the sample was set at 6 (402 nm) or 50 (505 nm) µW/cm^2^.

After sample illumination, MB-301 and MB-404 fluorescence emission was collected by the other fiber bundle bifurcation and collimated (Newport Corp., Model 77645). For the sake of simplicity, only one of the four detection channels is depicted in Fig. 3. MB-301 fluorescence was diverted 90° via a 567 nm long-pass dichroic mirror (ThorLabs DMLP567), passed through a 488 nm long-pass filter (Semrock LP02-488RE-25), a 15 nm bandpass filter centered at 540 nm (Semrock FF01-540/15-25), and detected by a Hamamatsu H7827-011 photomultiplier tube (PMT) with built-in amplifier. MB-404 fluorescence passed through the 567 nm long-pass dichroic mirror, a 532nm long-pass filter (Semrock LP03-532RE-25), a 40 nm bandpass filter centered at 593 nm (Semrock FF01-593/40), and detected by a Hamamatsu H7827-001 photomultiplier tube (PMT) with built-in amplifier. PMTs were powered by an Aligent E3630A power supply, and gains were adjusted via an inline potentiostat. Each amplified PMT output was synchronously detected by a lock-in amplifier (Stanford Research, SR830DSP), referenced to its respective source LED modulation frequency. Each lock-in amplifier output was read, displayed, and saved at a rate of 1 Hz using LabView software.

### 2.6. Transdermal fluorescence data analysis and calibration

Endogenous skin autofluorescence (baseline) contributed to the measured transdermal fluorescence signals for both the short and long wavelength measurements (MB-301 and MB-404 compounds respectively) and needed to be accounted for in relating the transdermal signals to agent concentrations. The process of gavaging the animals with these agents disturbed the optical measurements, resulting in a significant baseline shift on some of the animals. Therefore, rather than relying on the pre-gavage baseline measurements, the initial baselines were determined by back-extrapolating to the time zero gavage time, using a linear fit to the first five minutes of data collected after the animals had been returned to the anesthesia manifold. This initial (scalar) baseline was subtracted from the MB-404 fluorescence collected over the remainder of the experiment. The baseline-corrected MB-404 fluorescence remained well above zero at the end of the experiment (∼8 hours after gavage).

However, in the case of MB-301, scalar subtraction of the initial baseline resulted in the corrected fluorescence signal dropping below zero, starting at about 4 hours after gavage. This behavior is likely attributable to photobleaching of skin auto-fluorescence at the 400 nm excitation wavelength. Previous experiments have shown that skin photo-bleaching is highly wavelength dependent, with photo-bleaching increasing markedly with decreasing wavelength [28,29]. The final MB-301 baseline was estimated by fitting an exponential with a variable offset to the final 5.5 hours of the experiment, and using the fitted offset term as the final baseline value. A linearly varying baseline between the time of gavage and the end of the experiment was then used to correct the full MB-301 fluorescence time course.

After baseline correction, the data were smoothed and decimated by applying a 60-second wide trimmed mean filter, with exclusion of the lowest and highest quartiles of the sorted fluorescence values. The time scale of each channel (animal) was shifted so that time zero corresponded to the time of gavage.

**Fig. 4.**
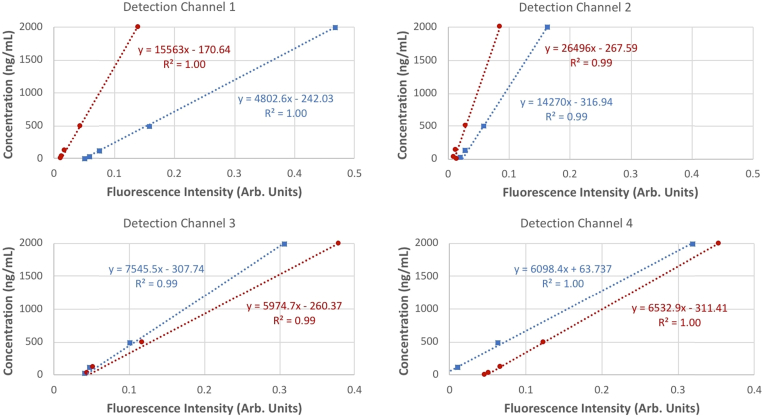
Calibration of MB-404 and MB-301 in 1% Intralipid across the 4 detection channels. Blue squares are the data points for the 400 nm excitation and the red circles are the data points for the 500 nm excitation. Dashed lines are linear regressions.

To correct for the differing optical properties of the two molecules and the variation in instrument sensitivity across the different instrument channels, a calibration procedure was developed. A series of solutions was prepared containing MB-301 and MB-404 at equal concentrations (0, 31.3, 125, 500, 2000 ng/mL), and 1% Intralipid to mimic skin light scattering properties. Standards were measured in each detection channel, using a 1 mm flow cell cuvette. Fluorescence signals for each standard were plotted against agent concentration to obtain calibration responses for each agent in each detection channel (Fig. 4

). The non-zero fitted intercept on each channel is indicative of a contribution to the signal due to leak-through of scattered excitation light. For the animal measurements, the signal contribution due to excitation leak-through was corrected along with skin auto-fluorescence by the baseline subtraction procedure described above, so only the fitted *in vitro* calibration slopes were used to estimate the *in vivo* agent concentrations. The substantial variation in slopes across the detection channels is due to variation in both optical alignment and in differences in amplifier gain settings.

Additional solutions containing only one or the other or both of the molecules were then used to assess the ability of the instrument to discriminate between the two molecules across the two excitation and emission arms of the instrument. The discrimination errors for the low (MB-301) and high (MB-404) molecular weight species, *E_L_* and *E_H_*, were defined as:

$$E_{L}=\;100*\frac{C_{L}(H+L)-C_{L}(L\;only)}{C_{L}(L\;only)}$$

$$E_{H}=\;100*\frac{C_{H}(H+L)-C_{H}(H\;only)}{C_{H}(H\;only)}$$

*C_L_* and *C_H_* are the concentration estimates of the low and high molecular weight species, respectively, as determined at the short (400 nm) and long (500 nm) excitation wavelengths. The discrimination errors above represent the concentration error induced by having both molecules present at the same time *(H + L)* compared to the concentration estimated when only the molecule of interest is present (*L only* or *H only*). Concentrations of 500 and 5000 ng/mL were employed respectively for the low (MB-301) and high (MB-404) molecular weight species. The results, summarized in Table 1

**Table 1.** Discrimination Error Estimates for Optically Determined Concentrations.

	Discrimination Error by Detection Channel			
	Channel 1	Channel 2	Channel 3	Channel 4
*E_L_*	−0.6%	−7.2%	5.2%	26%
*E_H_*	0.8%	−0.2%	−8.6%	10%

, show that the estimated discrimination errors were 10% or less, except for one short wavelength channel, where the discrimination error for MB-301 was 26%. The high variability in the discrimination errors across the instrument channels suggests that alignment sensitivity is critical, but that under optimal alignment (e.g. Channel 1), discrimination errors of less than 1% are achievable.

## 3. Results

### 3.1. Fluorophore recovery in urine results

The data shown in Table 2

**Table 2.** Parameters, analysis, and results of fluorophore recovery in urine samples. Blue indicates injured rats, red indicates control rats.

Rat Status	Rat Wt. (g)	Vol. Dosed (mL)	MB- 301 Dosed (mg)	MB- 404 Dosed (mg)	Urine volume (mL)	MB-301 in Urine (μg/mL)	MB-404 in Urine (μg/mL)	MB-301 in total urine volume (μg)	MB-404 in total urine volume (μg)
Rat 1	310	0.62	4.96	28.38	2.67	109.4	1318.9	291.5	3514.9
Rat 2	288	0.58	4.61	26.36	3.00	212.4	791.9	637.2	2375.7
Rat 3	308	0.62	4.93	28.19	2.30	217.8	231.5	500.9	532.5
Rat 4	286	0.57	4.58	26.18	1.95	110.0	133.0	213.9	258.7
Rat 5	340	0.68	5.44	31.12	2.45	167.2	579.7	409.7	1420.3
Rat 6	370	0.74	5.92	33.87	3.77	215.6	988.9	812.7	3728.2
Rat 7	388	0.78	6.21	35.52	2.00	176.2	135.4	352.3	270.8
Rat 8	324	0.65	5.18	29.66	2.18	279.8	130.5	608.5	283.8

contain the animal status (injured or control), the total volume of urine collected after eight hours, the concentration of MB-301 and MB-404 of dosing solution, the weight of each animal used for determining the volume of dosing solution, the concentration (µg/mL) of MB-301 and MB-404 measured by the HPLC analysis, and the total mass amount recovered in the urine.

**Fig. 5.**
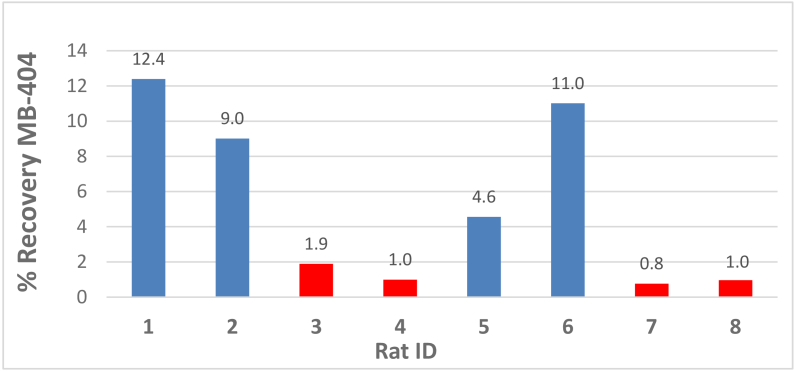
MB-404% recovery in Rat Urine. Blue bars are injured rats, red bars are control rats.

Bar graphs of the percent recovery in urine of MB-404 and MB-301 for injured and control animals are displayed in Figs. 5

and 6

**Fig. 6.**
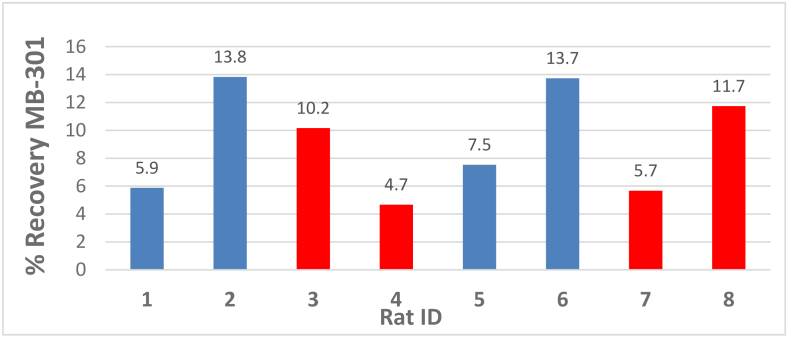
MB-301 recovery in Rat Urine. Blue bars are injured rats, red bars are control rats.

respectively. The percent recovery of MB-301 and MB-404 were calculated by dividing the total mass amount recovered in urine by the mass amount dosed.

Figure 5 clearly shows that the injured rats have more MB-404 collected in the urine than the control rats.

Figure 6 shows no definitive trend with respect to urine accumulation of MB-301 between the injured vs control rats.

Table 3

**Table 3.** Average (with standard deviation) percent recovery of fluorophore and p-value from two-sample t-test of injured to control.

Compound	Injured	Control	p-value (two-tail)
MB-301	10.2 +/- 4.1	8.1 +/- 3.4	0.45
MB-404	9.2 +/- 3.4	1.2 +/- 0.5	0.02

summarizes the average percent recovery in urine of MB-404 and MB-301 for the injured and control animals. The MB-404 recovery percentage was definitively greater for the indomethacin-injured cohort than for the control cohort. A two-sample t-test yields a two-tail p-value of 0.02, indicating a substantial difference between these two cohorts.

For MB-301, the average percent recovery between the injured and control cohorts is not as definitive. For this case, the two-sample t-test yields a two-tail p-value of 0.45, indicating essentially no difference between these two cohorts.

### 3.2. Transdermal fluorescence measurement analysis and results

**Fig. 7.**
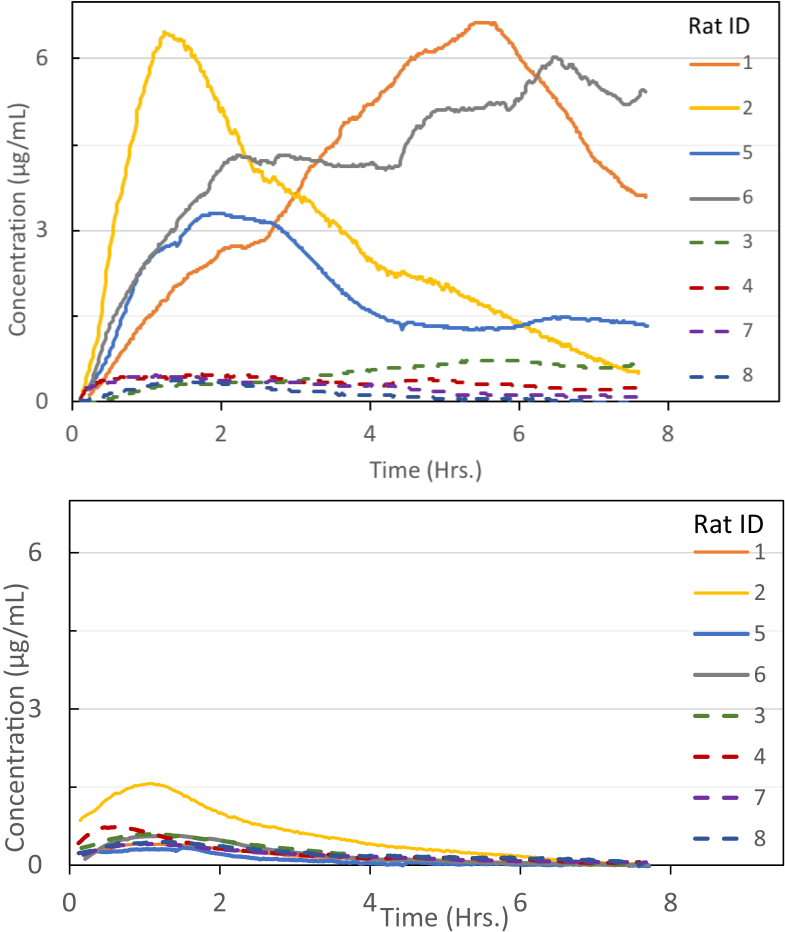
**(a).** Concentration time-course of MB-404 derived from transdermal fluorophore intensity measurements at 593 nm (with 505 nm excitation). Solid lines represent injured rats, dashed lines represent control rats. Time zero represents the time of gavage. **(b).** Concentration time-course of MB-301 derived from transdermal fluorophore intensity measurements at 540 nm (with 402 nm excitation). Solid lines represent injured rats, dashed lines represent control rats. Time zero represents the time of gavage.

The transdermal fluorescence measurements with baseline subtraction and conversion to concentration units from the calibration methodology are displayed in Fig. 7(a) and 7(b)

. The data are from two independent experiments with two injured and two control rats in each experiment. In Fig. 7(a), the MB-404 concentration time course for the injured animals clearly rises above the level of the control animals within half an hour after gavage. The time to peak concentration is highly variable among the treated animals, ranging from about one hour to greater than six hours. At eight hours following gavage, the MB-404 concentration for three of the four animals remains well above that of the controls.

In contrast, the MB-301 concentration time course plots, shown in Fig. 7(b), are highly overlapping among the injured and control animals, with the data for only one of the injured animals clearly distinct from the others. In all cases, for both injured and control animals, the MB-301 concentration peaked in under two hours relative to the time of gavage. And by the end of the eight-hour experiment, the MB-301 concentration has returned nearly to zero in all cases.

**Fig. 8.**
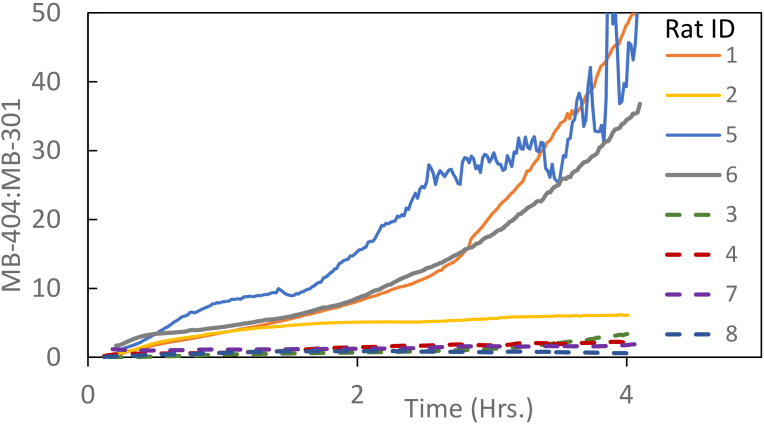
MB-404/MB-301 Ratio of concentration vs time. Solid lines represent the injured rats, dashed lines represent the control rats.

The common method of assessing gut permeability with sugar molecules is to plot the ratio of the concentrations of high to low molecular weight agents over time. A ratio of the high to low molecular weight fluorescent agents is shown in Fig. 8

. Here again, a clear separation between the injured and control rats is seen within a short (< 0.5 hour) time period. However, due to the rapid clearance of the MB-301 compound, the ratio becomes increasingly noisy with time, with large oscillations in some cases, as the concentration of the lower molecular weight species approaches zero.

**Fig. 9.**
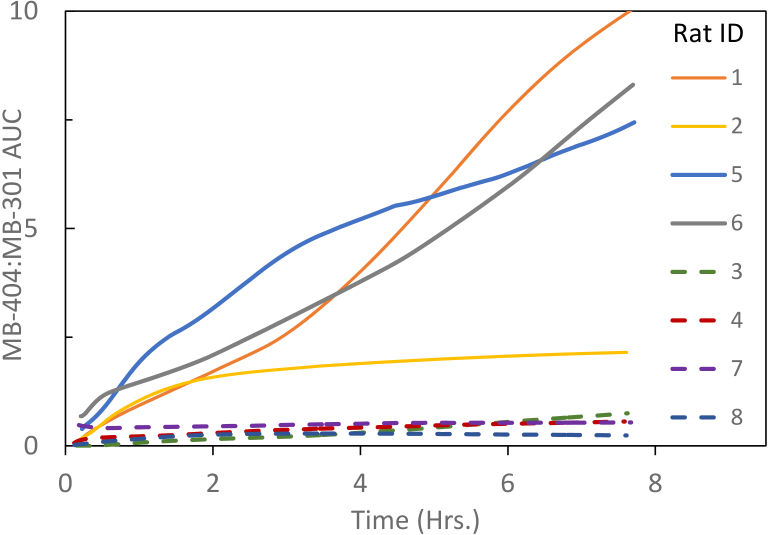
MB-404/MB-301 AUC Ratio of concentration vs time. Solid lines represent the injured rats, dashed lines represent the control rats. (The units for AUC for each compound are wt/vol * hour. The ratio is of course dimensionless.)

An analysis method to address the aforementioned issue is to compile the longitudinal area under the curve (AUC) for each compound. The AUC ratio of high to low molecular weight compounds is shown in Fig. 9

. This ratio remains stable and readily distinguishable between the injured and control rats throughout the course of the experiment. Similar to Fig. 7(a), the time course of the AUC ratio curves among the injured animals are highly variable, with the curve for one animal asymptotically approaching a ratio of about two, and the curves for the remaining three treated animals reaching between 7 and 10 by the end of the experiment, with little sign of leveling off. The AUC ratios for the control animals remained well below 1 throughout the experiment, and showed a much weaker time dependence.

## 4. Discussion

The average percent recovery of fluorophore in urine summarized in Table 2 strongly indicates that with indomethacin treatment, the larger molecule MB-404 permeates the gut membrane and enters the blood stream to a much greater extent than in rats challenged with vehicle alone, by nearly an order of magnitude. MB-301 permeates the gut membrane of injured and control rats to an equivalent extent. Thus the fluorophore ratio methodology essentially mimics the results of the dual sugar absorption test (DSAT) as initially hypothesized.

In a similar manner, the concentration estimates based on transdermal measurements of agent fluorescence clearly differentiated between the injured and control animals in the case of the high molecular weight species MB-404, but were less differentiated for the low molecular weight species MB-301. The added benefit of the transdermal measurements is that no sample collection was required and the differentiation could be observed within less than 30 minutes after the time of gavage.

These results naturally lead to the question of whether use of the low molecular weight agent is necessary in order to assess gut permeability. This topic will be explored in future studies. However, it should be pointed out that for humans in the general population the variability in gut transit times and the agent elimination rates from the blood stream by the kidney is likely to be much higher than the variability encountered between the laboratory-raised animals employed in these experiments. Assuming that a ratio of high to low molecular weight compounds is necessary to compensate for this variation, the AUC method offers the benefit of showing clear differentiation between injured and control animals within half an hour after gavage and extending over the entire duration of the experiment (approximately eight hours).

Another advantage of the ratiometric fluorescence technique employed here is that it can compensate for wavelength-independent variations in optical collection efficiency occurring over the course of the measurement, such as due to changes in probe angular positioning relative to the skin. However, wavelength-dependent variations in the local skin properties, such as those due to changes in local blood content, will not be corrected by measurement of fluorescence alone. The addition of diffuse reflectance measurements to assess tissue property variations in real time, therefore has the potential to improve the accuracy of the agent concentration estimates [30]. Whether such accuracy improvements would translate to meaningful differences in clinical assessment in GI permeability will be the subject of future experiments.

The variability in response across the four injured animals observed in this experiment may simply be an indication of the varying efficacy of indomethacin to induce gut injury due to biological variability of this animal model. The wide variation in time to peak concentration for the higher molecular weight compound may also be an indication of variation in the location of injury within the gut.

## 5. Conclusion

In our previous publication, the fluorophore technology was shown to be more sensitive than the DSAT method in the animal model for recovery of compounds in the urine (see Fig. 2 in Reference 18). In this paper, we extend that work to completely biocompatible fluorophores. Transdermal fluorescent detection of our dual fluorophore system mimics the DSAT results without requiring specimen collection or sophisticated chemical analysis. This technology for point-of-care determination of gut permeability should be easily adaptable to human use. A clinical study is expected to commence shortly.

## References

[1] Michielan A. D’Inca R., “Intestinal Permeability in Inflammatory Bowel Disease: Pathogenesis, Clinical Evaluation, and Therapy of Leaky Gut,” Mediators Inflammation 2015, 1–10 (2015). 10.1155/2015/628157 PMC4637104 26582965

[2] Smecuol E. Bai J. C. Vazquez H. Kogan Z. Cabanne A. Niveloni S. Pedreira S. Boerr L. Mauriño E. Meddings J. B., “Gastrointestinal permeability in celiac disease,” Gastroenterology 112(4), 1129–1136 (1997). 10.1016/S0016-5085(97)70123-9 9097995

[3] Johansson J. E. Ekman T., “Gut toxicity during hemopoietic stem cell transplantation may predict acute graft-versus-host disease severity in patients,” Dig. Dis. Sci. 52(9), 2340–2345 (2007). 10.1007/s10620-006-9404-x 17415646

[4] Nier A. Engstler A. J. Maier I. B. Bergheim I., “Markers of intestinal permeability are already altered in early stages of non-alcoholic fatty liver disease: Studies in children,” PLoS One 12(9), e0183282 (2017). 10.1371/journal.pone.0183282 28880885 PMC5589126

[5] Fotis L. Shaikh N. Baszis K. W. Samson C. M. Lev-Tzion R. French A. R. Tarr P. I., “Serologic Evidence of Gut-driven Systemic Inflammation in Juvenile Idiopathic Arthritis,” J. Rheumatol. 44(11), 1624–1631 (2017). 10.3899/jrheum.161589. 28916545 PMC5904838

[6] Teshima C. W. Goodman K. J. El-Kalla M. Turk S. El-Matary W. Valcheva R. Danchak R. Gordon M. Ho P. Mullins A. Wong D. Kao D. Meddings J. Huynh H. Dieleman L. A., “Increased Intestinal Permeability in Relatives of Patients With Crohn’s Disease Is Not Associated With Small Bowel Ulcerations,” Clin. Gastroenterol. Hepatol. 15(9), 1413–1418.e1 (2017). 10.1016/j.cgh.2017.02.028. 28286191

[7] Buhner S. Buning C. Genschel J. Kling K. Herrmann D. Dignass A. Kuechler I. Krueger S. Schmidt H. H Lochs H., “Genetic basis for increased intestinal permeability in families with Crohn’s disease: role of CARD15 3020insC mutation,” Gut 55(3), 342–347 (2006). 10.1136/gut.2005.065557 16000642 PMC1856071

[8] Peeters M. Geypens B. Claus D. Nevens H. Ghoos Y. Verbeke G. Baert F. Vermeire S. Vlietinck R. Rutgeerts P., “Clustering of increased small intestinal permeability in families with Crohn’s disease,” Gastroenterology 113(3), 802–807 (1997). 10.1016/S0016-5085(97)70174-4 9287971

[9] Kappelman M. D. Moore K. R. Allen J. K., “Recent Trends in the Prevalence of Crohn’s Disease and Ulcerative Colitis in a Commercially Insured US Population,” Dig. Dis. Sci. 58(2), 519–525 (2013). 10.1007/s10620-012-2371-5 22926499 PMC3576554

[10] Rubin D. T. Mody R. Davis K. L. Wang C. C., “Real-world assessment of therapy changes, suboptimal treatment and associated costs in patients with ulcerative colitis or Crohn’s disease,” Aliment. Pharmacol. Ther. 39(10), 1143–1155 (2014). 10.1111/apt.12727 24697826

[11] Shah S. Akbari M. Vanga R. Kelly C. P. Hansen J. Theethira T. Tariq S. Dennis M. Leffler D. A., “Patient perception of treatment burden is high in celiac disease compared with other common conditions,” Am. J. Gastroenterol. 109(9), 1304–1311 (2014). 10.1038/ajg.2014.29 24980880 PMC4159418

[12] Levesque B. G. Sandborn W. J. Ruel J. Feagan B. G. Sands B. E. Colombel J. F., “Converging goals of treatment of inflammatory bowel disease from clinical trials and practice,” Gastroenterology 148(1), 37–51.e1 (2015). 10.1053/j.gastro.2014.08.003 25127678

[13] Denno D. M. VanBuskirk K. Nelson Z. C. Musser C. A. Hay Burgess D. C. Tarr P., “Use of the lactulose to mannitol ratio to evaluate childhood environmental enteric dysfunction: a systematic review,” Clin. Infect. Dis. 59(suppl_4), S213–S219 (2014). 10.1093/cid/ciu541 25305289

[14] Sequeira I. R. Lentle R. G. Kruger M. C. Hurst R. D., “Standardising the lactulose mannitol test of gut permeability to minimise error and promote comparability,” PLoS One 9(6), e99256 (2014). 10.1371/journal.pone.0099256 24901524 PMC4047110

[15] Lostia A. M. Lionetto L. Principessa L. Evangelisti M. Gamba A. Villa M. P. Simmaco M., “A liquid chromatography/mass spectrometry method for the evaluation of intestinal permeability,” Clin. Biochem. 41(10-11), 887–892 (2008). 10.1016/j.clinbiochem.2008.03.016 18440311

[16] Lee G. O. Kosek P. Lima A. A. M. Singh R. Yori P. P. Olortegui M. P. Lamsam J. L. Oliveira D. B. Guerrant R. L. Kosek M., “Lactulose: mannitol diagnostic test by HPLC and LC-MSMS platforms: considerations for field studies of intestinal barrier function and environmental enteropathy,” J. Pediatr. Gastroenterol. Nutr. 59(4), 544–550 (2014). 10.1097/MPG.0000000000000459 24941958 PMC4222705

[17] Camilleri M., “Leaky gut: mechanisms, measurement and clinical implications in humans,” Gut 68(8), 1516–1526 (2019). 10.1136/gutjnl-2019-318427 31076401 PMC6790068

[18] Dorshow R. B. Hall-Moore C. Shaikh N. Talcott M. R. Faubion W. A. Rogers T. E. Shieh J. J. Debreczeny M. P. Johnson J. R. Dyer R. B. Singh R. J. Tarr P. I., “Measurement of gut permeability using fluorescent tracer agent technology,” Sci. Rep. 7(1), 10888 (2017). 10.1038/s41598-017-09971-y 28883476 PMC5589723

[19] Shiraia K. Yanagisawab A. Takahashib H. Fukunishia K. Matsuokac M., “Syntheses and fluorescent properties of 2,5-diamino-3,6-dicyanopyrazine dyes,” Dyes Pigm. 39(1), 49–68 (1998). 10.1016/S0143-7208(98)00008-4

[20] Rajagopalan R. Neumann W. L. Poreddy A. R. Fitch R. M. Freskos J. N. Asmelash B. Gaston K. R. Galen K. P. Shieh J. J. Dorshow R. M., “Hydrophilic pyrazine dyes as exogenous fluorescent tracer agents for real-time point-of-care measurement of glomerular filtration rate,” J. Med. Chem. 54(14), 5048–5058 (2011). 10.1021/jm200257k 21667980

[21] Davies N. M. Wright M. R. Jamali F., “Antiinflammatory drug-induced small intestinal permeability: the rat is a suitable model,” Pharm. Res. 11(11), 1652–1656 (1994). 10.1023/A:1018978308752 7870685

[22] Hesse C. Razmovski-Naumovski V. Duke C. C. Davies N. M. Roufogalis B. D., “Phytopreventative effects of Gynostemma pentaphyllum against acute Indomethacin-induced gastrointestinal and renal toxicity in rats,” Phytother. Res. 21(6), 523–530 (2007). 10.1002/ptr.2086 17380554

[23] Jacob M. Foster R. Sigthorsson G. Simpson R. Bjarnason I., “Role of bile in pathogenesis of indomethacin-induced enteropathy,” Arch. Toxicol. 81(4), 291–298 (2007). 10.1007/s00204-006-0149-2. 17151867

[24] Nishio H. Hayashi Y. Terashima S. Takeuchi K., “Protective effect of pranlukast, a cysteinyl-leukotriene receptor 1 antagonist, on indomethacin-induced small intestinal damage in rats,” Inflammopharmacology 15(6), 266–272 (2007). 10.1007/s10787-007-1585-1 18236018

[25] Sun Z. Lasson A. Olanders K. Deng X. Andersson R., “Gut barrier permeability, reticuloendothelial system function and protease inhibitor levels following intestinal ischaemia and reperfusion–effects of pretreatment with N-acetyl-L-cysteine and indomethacin,” Dig. Liver Dis. 34(8), 560–569 (2002). 10.1016/S1590-8658(02)80089-5 12502212

[26] Suzuki H. Hanyou N. Sonaka I. Minami H., “An elemental diet controls inflammation in indomethacin-induced small bowel disease in rats: the role of low dietary fat and the elimination of dietary proteins,” Dig. Dis. Sci. 50(10), 1951–1958 (2005). 10.1007/s10620-005-2967-0 16187203

[27] Wright M. R. Davies N. M. Jamali F., “Toxicokinetics of indomethacin-induced intestinal permeability in the rat,” Pharmacol. Res. 35(6), 499–504 (1997). 10.1006/phrs.1997.0194 9356198

[28] Debreczeny M. P. Bates R. Fitch R. M. Galen K. P. Ge J. Dorshow R. B., “Human skin auto-fluorescence decay as a function of irradiance and skin type,” Proc. SPIE 7897, Optical Interactions with Tissue and Cells XXII, 78971T (2011).

[29] Schleusener J. Lademann J. Darvin M. E., “Depth-dependent autofluorescence photobleaching using 325, 473, 633, and 785 nm of porcine ear skin *ex vivo*,” J. Biomed. Opt. 22(9), 091503 (2017). 10.1117/1.JBO.22.9.091503 28055059

[30] Bradley R. S. Thorniley M. S., “A review of attenuation correction techniques for tissue fluorescence,” J. R. Soc., Interface 3(6), 1–13 (2006). 10.1098/rsif.2005.0066 16849213 PMC1618480

